# Screening for Prognostic Biomarkers in Metastatic Adrenocortical Carcinoma by Tissue Micro Arrays Analysis Identifies P53 as an Independent Prognostic Marker of Overall Survival

**DOI:** 10.3390/cancers14092225

**Published:** 2022-04-29

**Authors:** Segolene Hescot, Matthieu Faron, Manal Kordahi, Christine Do Cao, Annabelle Naman, Livia Lamartina, Julien Hadoux, Sophie Leboulleux, Francois Pattou, Sébastien Aubert, Jean-Yves Scoazec, Abir Al Ghuzlan, Eric Baudin

**Affiliations:** 1 Department of Nuclear Medicine, Institut Curie, 92210 Saint Cloud, France; segolene.hescot@curie.fr; 2 Department of Surgery, Gustave Roussy, 94805 Villejuif, France; matthieu.faron@gustaveroussy.fr; 3 Department of Pathology, Gustave Roussy, 94805 Villejuif, France; manal_kordahi@hotmail.com (M.K.); jean-yves.scoazec@gustaveroussy.fr (J.-Y.S.); 4 Department of Endocrinology, Centre Hospitalier Universitaire Lille, 59000 Lille, France; christine.docao@chru-lille.fr; 5 Department of Endocrine Oncology, Gustave Roussy, 94805 Villejuif, France; a.naman@live.fr (A.N.); livia.lamartina@gustaveroussy.fr (L.L.); julien.hadoux@gustaveroussy.fr (J.H.); sophie.leboulleux@hcuge.ch (S.L.); eric.baudin@gustaveroussy.fr (E.B.); 6 Department of General and Endocrine Surgery, Centre Hospitalier Universitaire Lille, Université de Lille, 59000 Lille, France; pattou@univ-lille2.fr; 7 Institut of Pathology, Centre Hospitalier Universitaire Lille, 59000 Lille, France; sebastien.aubert@chru-lille.fr

**Keywords:** adrenocortical carcinoma, prognostic, tissue-micro-array, p53

## Abstract

**Simple Summary:**

The aim of our retrospective study was to identify and prioritize potential prognostic parameters in a well characterized metastatic ACC population. We identified for the first time P53 as an independent prognostic marker of metastatic adrenocortical carcinoma after mENSAT-GRAS parameter adjustment. This biomarker is easily available and should be considered in clinical practice together with Ki67 for the management of patient with advanced ACC. Moreover, this study underlies the importance of adjustment of potential biomarkers to validated prognostic factors in order to avoid the accumulation of invalidated biomarkers not usable in clinical practice.

**Abstract:**

Advanced adrenocortical carcinoma (ACC) has poor but heterogeneous prognosis. Apart from Ki67 index, no prognostic or predictive biomarker has been validated in advanced ACC, so far. We aimed at analyzing expression of a large panel of proteins involved in known altered pathways in ACC (cell cycle, Wnt/ß-catenin, methylation) to identify and prioritize potential prognostic or predictive parameters metastatic ACC population. We conducted a retrospective multicentric study. Overall survival (OS) and partial response according to RECIST 1.1 were primary endpoints. TMA was set up and 16 markers were analyzed. Modified ENSAT and GRAS parameters were characterized for prognostic adjustment. Results: We included 66 patients with a mean age at metastatic diagnosis of 48.7 ± 15.5 years. Median survival was 27.8 months. After adjustment to mENSAT-GRAS parameters, p53 and PDxK were prognostic of OS. No potential biomarker has been identified as predictive factor of response. We identified for the first time P53 as an independent prognostic marker of metastatic adrenocortical carcinoma after mENSAT-GRAS parameter adjustment. Prognostic impact of Wnt/ß-catenin alterations was not confirmed in this cohort of metastatic ACC.

## 1. Introduction

Adrenocortical carcinoma (ACC) is a rare cancer originating from the adrenal cortex with an incidence of less than 0.7–1.5 per 1 million people per year [1]. Its prognosis is poor: almost 50% of ACC are metastatic at initial diagnosis and when localized, the risk of recurrence is high especially in case of Ki-67 index > 10% [2,3,4,5,6]. The median overall survival (OS) of metastatic ACC patients varies between 10 and 20 months with a 5-year survival around 10% [1]. However, the prognosis is variable and long survivors have been recently described [7,8]. Nowadays, prognostic factors of advanced ACC are clinical and pathological parameters [8]. Recently, the new mENSAT TNM classification combined with GRAS parameters (Grade defined by Weiss score below or above 6 or Ki-67 below or above 20%; R0 resection status; age below or above 50 years; tumor- or hormone-related symptoms) was shown to allow the best risk stratification in term of OS in stage III–IV ACC patients [9]. No prognostic molecular marker has emerged aside from Ki-67 based on mENSAT-GRAS adjustment [10,11,12].

Different behavior of advanced ACC in term of survival suggests a different biology. Several recent-omics studies have highlighted molecular pathways involved in ACC tumorigenesis and attempted to identify a prognostic role of molecular classifications [10,13]. Hypermethylation appears to be associated with increased aggressiveness and a signature of the methylation status of 4 genes (*PAX5*, *PAX6*, *PYCARD* and *GSTP1*) was shown to correlate to OS independently of ENSAT stage and Ki-67 [14]. Pangenomic studies have identified genetic alterations in 50% of ACC, the most frequent genes involved belonging to cell cycle and Wnt-β-catenin pathways [15,16,17]. Their prognostic impact has been suggested in many studies but neither validated in an independent research laboratory nor validated against the most accurate clinicopathological classifications, namely mENSAT-GRAS.

From a methodological standpoint, simple and robust methodology applicable in every specialized center is needed. Immunohistochemistry for protein expression analysis can therefore be considered as a relevant tool. Historically, based on ACC-related inherited syndrome, β-catenin and p53 have been evaluated by β-catenin nuclear staining or aberrant p53 expression using immunochemistry in ACC patients. These alterations were classified as having a prognostic role but not validated as independent prognostic factors with respect to mENSAT-GRAS criteria [18]. During the last decade, many molecular candidates with a potential prognostic impact have emerged, mostly studied one after the other without prioritization, but no single one is currently validated as mentioned in the most recent guidelines [19]. In the same manner, several predictive factors of response to mitotane and platinum-based therapy have been proposed but their validation is still pending [20,21,22,23].

Therefore, we studied expression of a large set of relevant target protein in tissue micro arrays (TMA) issued from a large and well characterized cohort of metastatic ACC patients with the aim to correlate their expression to OS and response to treatment and to prioritize their use in routine practice.

## 2. Materials and Methods

### 2.1. Clinical Data

Inclusion criteria were histologically proved stage IV ACC with tissue available for TMA analysis. In this case, 66 adult patients followed in two centers (CHU Lille and Gustave Roussy), were selected for the study. An informed consent was obtained from all patients. The medical file of each patient was reviewed by one investigator (SH) to record all clinical parameters at the time of metastasis diagnosis and sample collection, as well as data about treatment outcomes including response to therapy according to RECIST 1.1 criteria [24]. The description and cutoff values of each parameter are given in Table 1. Cutoff values were chosen in order to limit the number of subgroups considering the size of the cohort. In this case, 55 samples originated from primary tumor while 11 were from metastasis when the primary was not available in the same way as previous [10,25]. For 2 patients, matched primary-metastasis samples were available and no difference was found. Chemotherapy was administrated before time of sampling in 4 patients. The study was approved by Gustave Roussy ethical committee.

### 2.2. Selected Biomarkers

Proteins were selected according to literature data, as follows:

Proteins involved in main altered pathways in ACC pathogenesis, including:

- cell cycle: p16, p53, Rb, ATM, [26]-Wnt-β-catenin: β-catenin, LEF1 pathway [27],
- others proteins involved in adrenal steroidogenesis or tumorigenesis: GATA6 [28], SF1 [29],
- methylation markers: MGMT [30], PAX6, GSTP1 [14].

Potential predictive factors of response, to platinum-based chemotherapy including: PDxK [31] or, to mitotane including: RMM1 [20], SOAT1 [23], TSPO [21] and FATE1 [22] or to immunotherapy: PDL1.

Their physiological role and potential role in ACC are detailed in Table 2.

### 2.3. Tissue Microarrays Construction

Tissue microarrays (TMA) were prepared by the Laboratory of Experimental and Translational Pathology (PETRA), Gustave Roussy Cancer Campus from selected tissue material. All the H&E slides from the 66 cases were examined by two pathologists (AA and MK) for diagnosis confirmation. In each case, one representative slide was selected and marked for two areas with high tumor cellularity.

Three punches of 1 mm in diameter from each block were obtained to avoid bias due to tumor heterogeneity, randomly distributed in the recipient block. In this study, four TMA of 27 to 122 samples each were prepared.

### 2.4. Immunohistochemistry

Immunohistochemical techniques were carried out by the Laboratory of Experimental and Translational Pathology (PETRA), Gustave Roussy Cancer Campus. Staining platforms, antibody clones, dilutions and the pattern of the staining are detailed in supplemental Table S1. Expression of each protein was analyzed by qualitative staining (expression or absence of expression) for Rb, LEF1, SF1, GSTP1, PDxK and FATE1, (overexpression, i.e., expression of 100% of cells) for p53 and p16, localization of staining (presence of nuclear staining of 100% of cells) for beta-catenin or quantitative staining (H-score, mean of 3 samples) for ATM, GATA6, MGMT, PAX6, RRM1, SOAT1 and TSPO. These methods were determined depending on protein function and literature results and are detailed in Table 2 ([32]) Cut-off for H-score was homogeneously determined at 150 for each relevant protein considering the repartition of their expressions patterns (similar to medians). Different type of staining patterns for all protein are provided in Figure S1.

### 2.5. Statistical Analysis

Quantitative variables were presented as mean (standard deviation (sd)) and qualitative variables as count (percentage). Overall survival, as primary endpoint, was calculated according to the Kaplan-Meier method from the time of metastatic diagnosis to the date of death from any cause. Univariable analysis used a single variable Cox proportional hazard model. Any protein achieving a *p* < 0.05 in the univariable model was subsequently tested in a multivariable model with the other significant proteins. A prespecified multivariable model adjusting the significant proteins between them and for the mENSAT-GRAS criteria in order to evaluate the added prognostic value of new putative parameter and to prioritize.

Response to therapy were tested by binary logistic regression according to RECIST 1.1 and long term survival. Long term survival was defined as an overall survival longer than 24 months and considered as a binary variable as no patient was censored before 24 months. Association between proteins and long terms survivor was evaluated with a single variable binary logistic model.

## 3. Results

### 3.1. Clinicopathological Characteristics

The clinical and pathological characteristics of the 66 patients are summarized in Table 1. All patients in our cohort were metastatic, 36.4% with synchronous metastasis and 40.9% had three or more metastatic organs. All patients were characterized according to mENSAT-GRAS criteria including mean age at metastatic disease of 48.70 ± 15.5 years and hormonal secretion present in 38 patients (58.5%). Weiss score was above 6 in 36 (67.9%) cases. Ki67 index was higher than 20% in 21 (32.3%) cases. There was no oncocytic ACC in the cohort.

### 3.2. Expression Profile of Biomarkers

**Figure 1:**
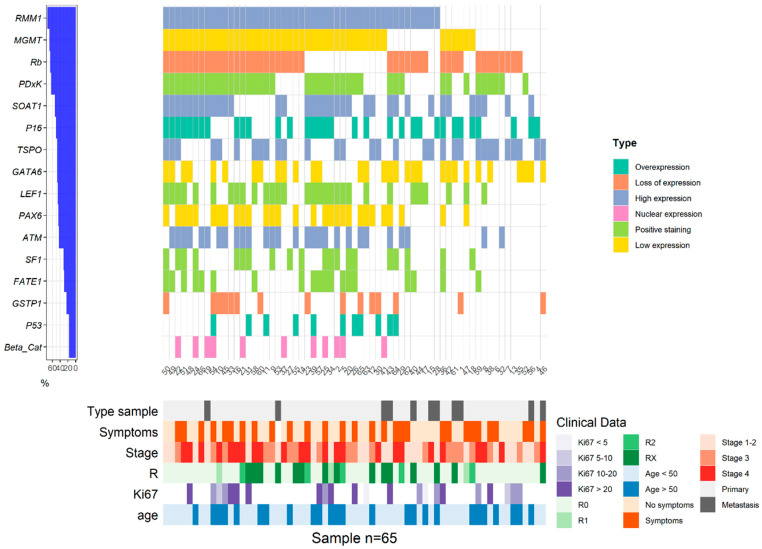
Expression profile of biomarkers according to clinico-pathological criteria.

**Figure 2:**
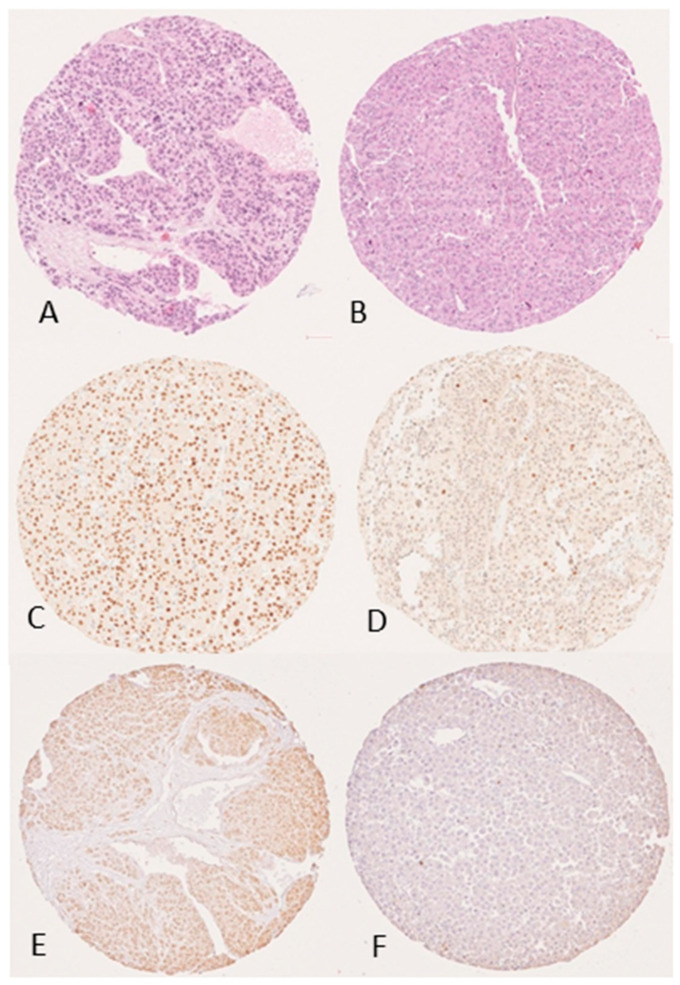
P53 and PDXk expression in ACC. (**A**,**B**) Hematoxylin and eosin (H&E) stains (10×). (**C**–**F**) Immunostaining (10×); (**C**) P53 overexpression staining pattern; (**D**) Wild type staining of P53 (characterized by an admixture of negative cells, weakly and strongly positive cells); (**E**) immunopositive tumor for PDxK; (**F**) immunonegative tumor for PDxK.

All expression profiles are described in Table 2 and detailed according to clinicopathological criteria in Figure 1. P53 was overexpressed in 11 tumors (16.9%; Figure 2); Rb was lost in 9 (17.3%). P16 was overexpressed in 33 (50.6%) from which 5 were Rb negative. At least one biomarker of the cell cycle was altered in 95.4% of patients. These expression profiles were not mutually exclusive. A nuclear expression of β-catenin, as a marker of Wnt-β-catenin pathway activation, was described in 11 cases (16.9%). LEF1 was expressed in 30 cases (46.2%). At least one biomarker of the Wnt-β-catenin pathway was altered in 49.2% of patients. Mutual P53 overexpression and nuclear expression of β-catenin were associated in 3 cases. No correlation was found between abnormalities in cell cycle proteins (p53, Rb/p16, ATM) and Wnt/β-catenin pathway (β-catenin, LEF1). GATA6 expression was low in 34 cases (52.3%) while SF1 was expressed in 20 cases (30.3%). MGMT expression was low in 22 patients (33.3%). GSTP1 expression was lost in 49 cases (76.6%) and PAX6 expression was low in 35 cases (54.7%). At least one biomarker of methylation was altered in 84.8% of patients (Figure 1).

About potential predictive markers of response to therapy, PDxK, RRM1, SOAT1, TSPO and FATE1 expressions were high in, respectively, 63.1%, 72.3%, 53.8%, 49.2% and 29.2% of samples (Figure 2). Of note, no expression of PDL1 was found in our cohort.

### 3.3. Prognostic Value

**Figure 3:**
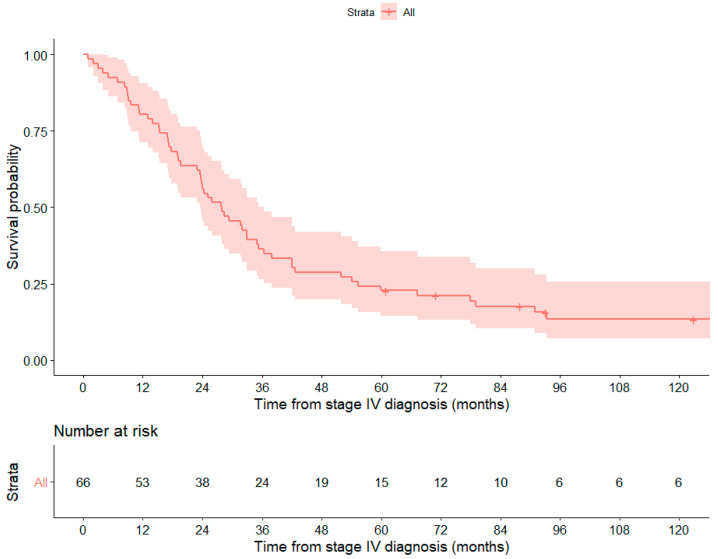
Overall Survival from date of metastatic diagnosis in the cohort.

**Figure 4:**
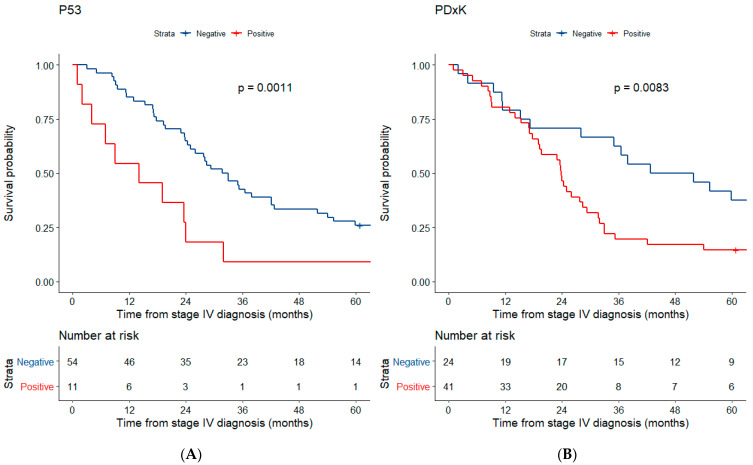
Survival curves of univariable analysis. (**A**) P53 and (**B**) PDxK.

Median overall survival (OS) from time of metastatic diagnosis was 28 months [23.5–36.5] and 1-year survival and 5 year-survival were 80.3 and 22.7%, respectively (Figure 3). In this case, 38 patients (58%) were alive at 24 months and therefore considered as long survivors. In univariable analysis, overall survival from the time of metastatic disease was statistically associated with the expression pattern of p53, GSTP1, PDxK, FATE1 and RMM1 while in multivariable analysis PDxK and GSTP1 expression remained significantly associated with worst prognosis (Table 3). When adjusted to mENSAT GRAS validated prognostic markers, p53 and PDxK positive staining were independently associated to overall survival with an Odds Ratio of 2.24 and 2.73, respectively (Table 3; Figure 4). Moreover, overexpression of p53 staining (*p* = 0.021) and TSPO level of expression (*p* = 0.0071) were significantly lower in long survivors (defined by an overall survival > 24 months).

### 3.4. Predictive Markers of Response to Therapy

All patients received mitotane at the time of metastasis with median treatment duration of 28.5 ± 34.8 months. Of them, 23 received mitotane prior to recurrence. Plasma mitotane levels were available in 53 patients and reached 14 mg/l in 86.8% of them. Here, 52 patients (78.8%) received platinum-based chemotherapy associated or not to mitotane. In this case, 50 out of 66 patients had RECIST 1.1 evaluable disease; the others received locoregional treatments of all targets or died before first evaluation. Best response according to RECIST 1.1 criteria was partial response or stable disease for 17 (34%) and 16 (32%) of cases, respectively. In this case, 17 patients (34%) had progressive disease whatever the line of treatment. No biomarker was found to be significantly associated with response to treatment according to RECIST 1.1. Mitotane duration and plasma levels were predictive of best response.

## 4. Discussion

Here, 16 putative biomarkers were analyzed in a large series of 66 metastatic ACC. The aim of our study was to identify and prioritize independent prognostic biomarkers through their immunohistochemical pattern of expression, which could be easily used in the diagnostic setting in all centers. To that end, TMA of a large cohort of patients with metastatic ACC was studied for a large set of biomarkers analyzed at three same times with an appropriate mENSAT-GRAS criteria characterization. This strategy is complementary to the “one after the other” evaluation of single biomarker inconsistently adjusted for most relevant clinical prognostic. The overall survival of the cohort from the time of metastatic disease was quite high (median 28 months) allowing the analysis of long term survivors. This long term OS may be explained by the selection of patients with available tissue of the primary tumor most frequently achieved in good prognostic advanced ACC. Mirroring the long term OS, the rate of response was at the upper range of the literature. Our study identified for the first time P53 as the strongest prognostic molecular biomarker, independent prognostic marker of metastatic adrenocortical carcinoma after mENSAT-GRAS parameter adjustment.

Relevant biomarkers were selected according to the main altered pathways in ACC pathogenesis discovered in previous studies. As compared to, the expression of biomarkers such as P53, Rb, GATA6 or SOAT1 [18,23,33,34] was consistent with that reported in previously published data except from SF1 that was less often positive in our cohort than in others [29,34]. Some previous studies suggested that cell cycle abnormalities at the protein level in ACC were associated with poor prognosis [18,33,35]. We confirm in this study that P53 overexpression is a factor of poor prognosis and demonstrate for the first time that it is an independent prognostic marker in a multivariate analysis adjusted for mENSAT-GRAS criteria. Neither other cell cycle-related protein expression pattern nor the alteration of the cell cycle pathway as a whole (by any altered protein studied) provided added prognostic value. On the other hand, the prognostic relevance of β-catenin expression is not validated in this cohort and no added prognostic value of LEF1 or the combined alteration of both proteins could be identified neither. Discrepant results may be explained by a different subgroup analysis since our study was performed in metastatic ACC specifically, with an OS calculated from the time of metastatic disease diagnosis.

Jouinot et al. have shown that hypermethylation provide prognostic information that remains significant after grade adjustment in stage I–IV ACC patients [14]. Their data were based on the evaluation of the methylation status by methylation-specific multiplex ligation-dependent probe amplification (MS-MLPA) in a panel of 4 genes. In our study, protein expression of 3 known validated epigenetic-targets was used. With this methodology, we found a correlation between low GSTP1 expression and overall survival. However, no biomarker of hypermethylation is prognostic in our multivariable analysis after mENSAT-GRAS adjustment and therefore suitable for clinical prognostic use. Positive expression of PDxK (involved in vitamin B-related metabolic processes) was described for the first time as a potential prognostic parameter that warrants further validation. Indeed, its expression is negatively correlated to survival in lung cancer [31].

PDxK and GSTP1 expressions have been previously correlated to resistance to platinum-based chemotherapy in ovarian and esophageal carcinomas [36,37]. However, the negative expression of these two markers is not significantly associated with response to platinum in our cohort. These results may be explained by the low number of response but also by the fact that tumor responses may not be strictly related to platinum-based chemotherapy but also due to mitotane combined treatment. As a surrogate marker, we looked for prognostic factors of long term survival (>24 months) and identified a significant association at univariable level with absence of p53 overexpression and low TSPO expression. Further studies should confirm the hypothesis of their role as predictive markers of response to platinum-based therapy or mitotane. Only mitotane duration and plasma levels were predictive of best response. We failed to identify potential predictive markers of response to Mitotane. That might be also explained by a lack of power. However, as recently published in a larger ENSAT cohort, SOAT1 is not predictive of response to mitotane [23], neither other candidates such as RRM1were found to correlate to tumor response. Evaluation of response to mitotane remains challenging because of its delayed response pattern and potential association to chemotherapy [7,38]. No predictive factor is validated to date for metastatic ACC and further studies are needed [39].

Our study has some limitations: it is retrospective; the use of TMA has the interest to make it possible to study simultaneously a large number of samples, but because of small size of the cores, sample bias (including tumor heterogeneity) might be higher than in the study of whole sections. Samples include primary and metastasis. However, results of the two patients with both primary and metastatic available tissues did not show any additional molecular event. Moreover, we have noticed the same limitation in recent remarkable manuscripts (ref [11] Mohan et al.). As most of retrospective prognostic studies, impact of treatments is not taken into account for the prognostic analysis. In addition, as for all similar studies that aim at looking for predictive markers of response in ACC, the response to mitotane is difficult to describe. In accordance with recent publications, the study could be extended to other potential prognostic factors such as VAV2 [25], TERT [40], EZH2 [41], FSCN1 [42], GoS2 [11] or other markers of senescence (P21, phosphor-H2AX). However, none of this biomarker was validated in metastatic ACC specifically after adjustment to mENSAT-GRAS parameters. Moreover, in contrast with previous microarrays studies, this one is the first that includes a comprehensive clinical and pathological characterization that focus on metastatic ACC and allows a multivariate analysis with mENSAT-GRAS criteria [43]. Finally, last limitation is that no genomic profiling data are available in this adult cohort.

## 5. Conclusions

To conclude, we identified for the first time P53 as an independent prognostic marker of metastatic adrenocortical carcinoma after mENSAT-GRAS parameter adjustment. This biomarker is easily available and should be considered in clinical practice together with Ki67 for the management of patient with advanced ACC. Moreover, most of the previously potential prognostic parameters are not validated in our study. Our study underlies the importance of adjustment of potential new biomarkers to validated prognostic factors in well-defined population of ACC patients regarding their TNM stage in order to avoid the accumulation of invalidated redundant biomarkers providing no additional information in clinical practice.

## Figures and Tables

**Table 1:** Clinicopathological characteristics.

Variable	Category	N Evaluable	Total N (%)
**At initial diagnosis**			
Age		66	47.7 (±15.5) years
Sex Ratio		66	23 M/43F
Stage	I–II	66	22 (33.3)
	III		20 (30.3)
	IV		24 (36.4)
Tumoral syndrome	Yes	65	36 (55.4)
Hormonal secretion	Yes	65	38 (58.5)
Tumor size	≤10 cm	64	26 (40.6)
	>10 cm		38 (59.4)
Weiss score	3 to 5	53	17 (32.1)
	6 to 9		36 (67.9)
Ki67	≤20	65	44 (67.7)
	>20		21 (32.3)
Resection status	R0 R1/R2	51	42 (84.3) 9 (15.7)
**At metastatic diagnosis**			
Age		66	48.7 (±15.5) years
Symptoms		65	38 (58.5)
Stage	IVA IVB IVC	66	22 (33) 17 (26) 27 (41)
**Treatments**			
Mitotane duration (months)		60	28.5 (±34.8)
Mitotane > 14 mg/L		53	46 (86.8)
Treated with platinum	Yes	66	52 (78.8)
Best response	CR/PR	50	17 (34)
	SD		16 (32)
	PD		17 (34)
Disease control > 12 months	Yes	50	23 (34.8)

CR: complete response; PR: partial response, SD: stable disease; PD: progression disease.

**Table 2:** Analysis of selected biomarkers.

Protein	Function/Pathway	Relevant Pattern	Potential Role in ACC	*n* (%)
**P53**	tumor suppressor/cell cycle	Overexpression	Prognostic	11 (16.9)
**P16**	tumor suppressor/cell cycle	Overexpression	Prognostic	33 (50.8)
**Rb** (retinoblastoma)	tumor suppressor/cell cycle	Loss of expression	Prognostic	9 (17.3)
**ATM** (ataxia telangiectasia mutated)	kinase activated by DNA double-strand breaks	High expression *	Prognostic	28 (43.1)
**ß-catenin**	intracellular signal transducer/Wnt-pathway	Nuclear expression (activation)	Prognostic	11 (16.9)
**LEF1** (lymphoid enhancer-binding factor 1)	transcription factor, downstream mediator/Wnt-pathway	Positive staining	Prognostic	30 (46.2)
**GATA6** (GATA-binding protein 6)	transcription factor/adrenal steroidogenesis	Low expression *	Prognostic	34 (52.3)
**SF1** (Steroidogenic factor 1)	transcription factor/adrenal development	Positive staining	Prognostic	20 (30.3)
**MGMT** (O^6^-alkylguanine DNA alkyltransferase)	DNA repair protein	Low expression *	Prognostic	22 (33.3)
**PAX6** (Paired box protein 6)	transcription factor/encoded by hypermethylated gene	Low expression *	Prognostic	35 (54.7)
**GSTP1** (Glutathione S-Transferase Pi 1)	enzyme/detoxification/encoded by hypermethylated gene	Loss of expression	Prognostic	49 (76.6)
**PDxK** (Pyridoxal kinase)	vitamin B-related metabolic processes	Positive staining	Predictive of response to platin	41 (63.1)
**RRM1** (Ribonucleotide Reductase Catalytic Subunit M1)	enzyme/production of deoxyribonucleotide	High expression *	Prognostic and predictive of resistance to mitotane	47 (72.3)
**SOAT1** (Sterol O-Acyltransferase 1)	adrenal steroidogenesis, potential target of mitotane	High expression *	Potential target of mitotane	35 (53.8)
**TSPO** (Translocator protein)	adrenal steroidogenesis, potential target of mitotane	High expression *	Potential target of mitotane	31 (49.2)
**FATE1** (Fetal and Adult Testis-Expressed 1)	encoded by a gene targeted by SF1	Positive staining	Predictive of response to mitotane	19 (29.2)

* defined as H-Score > 150 (high) or <150 (low).

**Table 3:** Univariate and multivariate analysis of protein expression as prognostic factors of overall survival without and with adjustment for mENSAT GRAS.

Variable	Category	HR	*p*	HR	*p*	HR	*p*
		Univariate		Multivariate		Multivariate (adjusted for mENSAT GRAS)	
P53	Negative	1	**0.0011**	1	0.19	1	**0.048**
	Positive	2.93 [1.49–5.75]		1.69 [0.8–3.56]		2.24 [1.05–4.74]	
PDxK	Negative	1	**0.0083**	1	**0.024**	1	**0.0027**
	Positive	2.14 [1.2–3.81]		2.11 [1.09–4.09]		2.73 [1.38–5.37]	
GSTP1	Negative	1	**0.028**	1	**0.019**		
	Positive	1.96 [1.06–3.62]		2.27 [1.19–4.32]			
FATE1	Negative	1	**0.04**	1	0.14		
	Positive	1.82 [1.02–3.26]		1.61 [0.86–3.01]			

## Data Availability

The data presented in this study are available in this article and supplementary material.

## Supplementary Materials

The following supporting information can be downloaded at: https://www.mdpi.com/article/10.3390/cancers14092225/s1, Figure S1: Representative pictures of immunohistochemical staining of different markers. 1. overexpressed ATM in 100% of cells. 2. ATM with heterogeneous expression (20% strong, 20% weak and 60% moderate. H-score: 200). 3. positive Beta-cat with nuclear and cytoplasmic staining. 4. negative Beta-cat with membranous normal staining. 5. heterogeneous weak staining of Fat1. 6. high expressed Fat1 (strong cytoplasmic staining in 100% of cells). 7. positive nuclear staining of Gata6. 8. negative Gata6. 9. moderate cytoplasmic staining of GSTP1. 10. negative GSTP1. 11. Ki67 positive in 10% of cells. 12. Ki67 positive in 90% of cells. 13. Lef1 positive in 100% of cells. 14. heterogeneous expression of Lef1. 15. positive MGMT with nuclear staining (5×). 16. negative MGMT, see nuclear staining in normal endothelial cells. 17. overexpressed P16 with strong nuclear and cytoplasmic staining in 100% of cells. 18. negative P16. 19. wild type staining of P53 characterized by an admixture of negative cells, weakly and strongly positive cells. 20. positive overexpressed P53; strong staining in 100% of cells. 21. positive Pax6 with moderate staining. 22. strongly expressed Pax6. 23. conserved expression of Rb. 24. loss of expression of Rb, see positive endothelial normal cells. 25. strongly expressed RMM1. 26. moderately expressed RRM1. 27. positive SF1 with nuclear staining. 28. loss of staining of SF1. 29. SOAT1: weakly positive in few cells. 30. highexpressed pattern of SOAT1. Table S1: Protein analysis: reference and methods of analysis.
