# Supplementary material for: Screening for Prognostic Biomarkers in Metastatic Adrenocortical Carcinoma by Tissue Micro Arrays Analysis Identifies P53 as an Independent Prognostic Marker of Overall Survival
Source: Cancers (Basel). 2022 Apr 29;14(9):2225. doi: 10.3390/cancers14092225 (PMC9099575; doi:10.3390/cancers14092225)
Supplement: Supplementary file 1 [file cancers-14-02225-s001.zip › supplementary material-final/Table S1.pdf]

Table S1. Protein analysis: reference and methods of analysis.

Antibody	Clone	Company	Reference	Dilution	Staining pattern
**Ki67**	Mouse MIB1	DAKO	M7240	1 : 50	Nuclear
**P16**	Mouse	Roche	805-4713	Pre-diluted	Nuclear
**P53**	Mouse DO7	DAKO	M7001	1 : 20	Nuclear
**Rb**	D20B12	Leica	NCL-L-RB-358	1 : 100	Nuclear
**ATM**	Mouse Y170	Abcam (Epitomics)	ab32420	1 : 50	Nuclear, cytoplasmic and membranous
**B-catenin**	Mouse bcat 1	DAKO	M3539	1 : 70	Nuclear, cytoplasmic and membranous
**LEF1**	EPR2029Y	abcam	ab137872	1 : 100	cytoplasmic
**GATA6**	Rabbit	Santa Cruz	sc9055	1 : 200	Nuclear
**SF1**	Mouse	Thermo scientific	434200	1 : 100	Nuclear
**FATE1**	Mouse	Abcam	ab57842	3 µg/ml	Cytoplasmic and membranous
**MGMT**	Mouse MT3.1	Thermo scientific	MA5 13506	1 : 50	Nuclear
**PAX6**	Mouse AD2.38	Abcam	ab78545	1 : 150	Nuclear
**GSTP1**	Mouse 353-10	LabVision	MS-1828-S1	1 : 50	Nuclear and cytoplasmic
**PDxK**	Polyclonal	Interchim	AP7167a	1 : 25	cytoplasmic
**RRM1**	Poly	Proteintech	10526-1-AP	1 : 50	Nuclear and cytoplasmic
**SOAT1**	Rabbit	Abcam	ab39327	1 : 1000	Cytoplasmic
**TSPO**	Mouse EPR5384	Abcam	ab109497	1 : 100	Cytoplasmic and membranous
