# Supplementary figures and images for: Screening for Prognostic Biomarkers in Metastatic Adrenocortical Carcinoma by Tissue Micro Arrays Analysis Identifies P53 as an Independent Prognostic Marker of Overall Survival
Source: Cancers (Basel). 2022 Apr 29;14(9):2225. doi: 10.3390/cancers14092225 (PMC9099575; doi:10.3390/cancers14092225)

## ATM

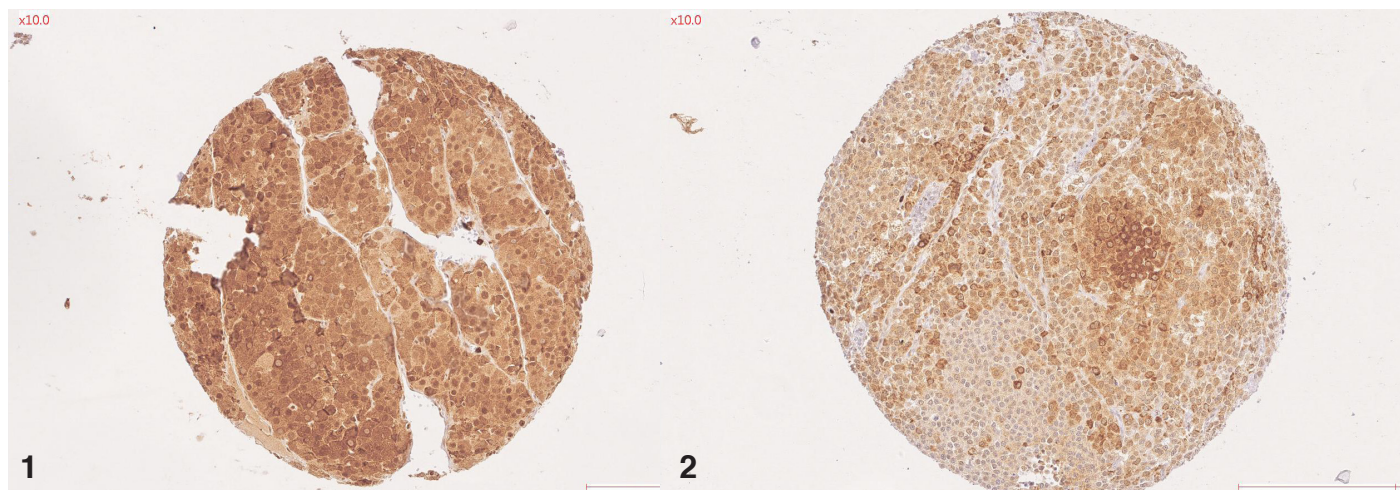

## Bcat

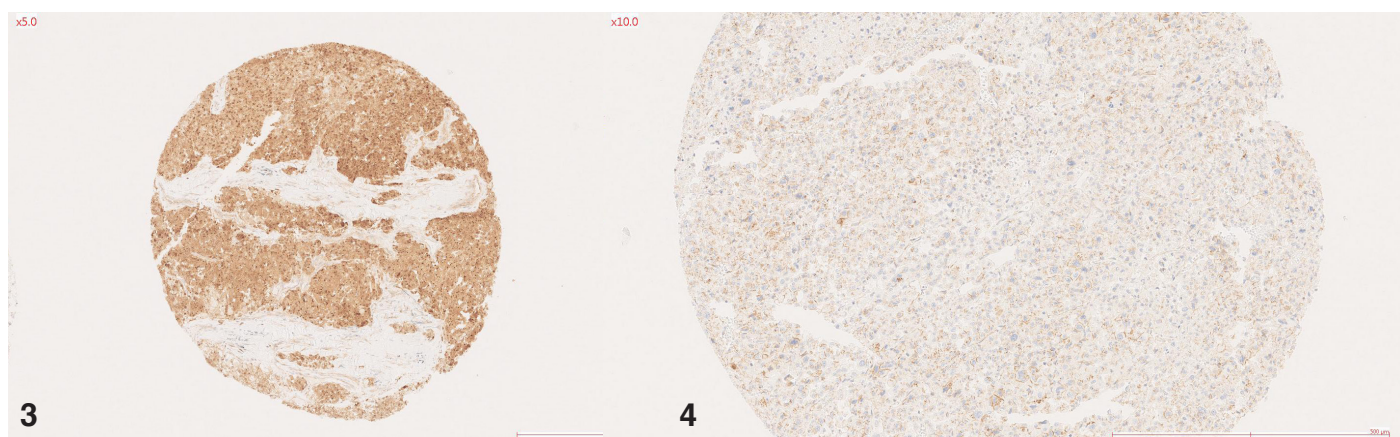

## FATE1

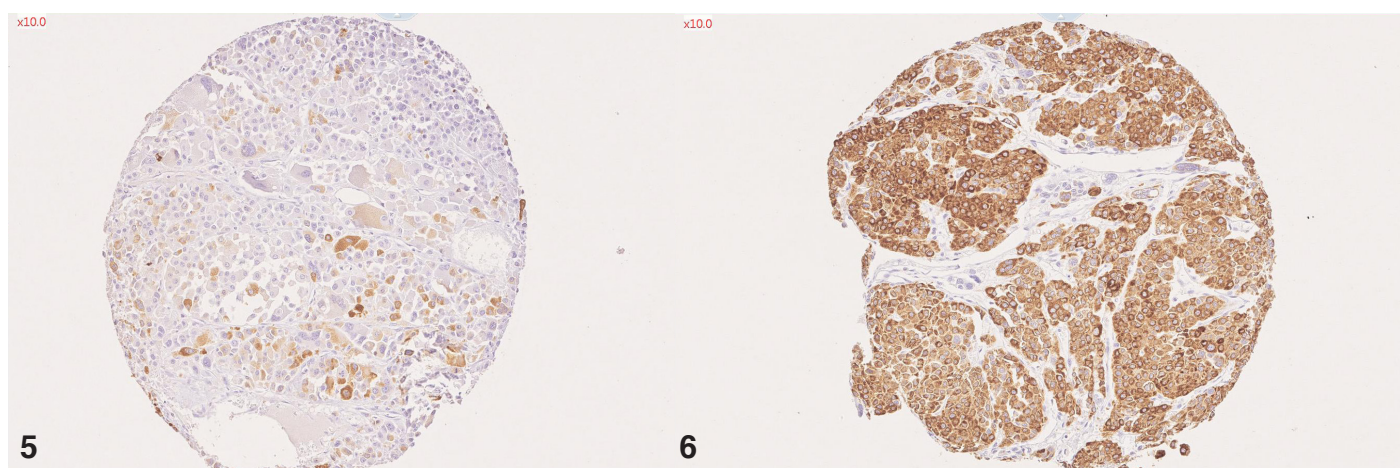

## GATA6

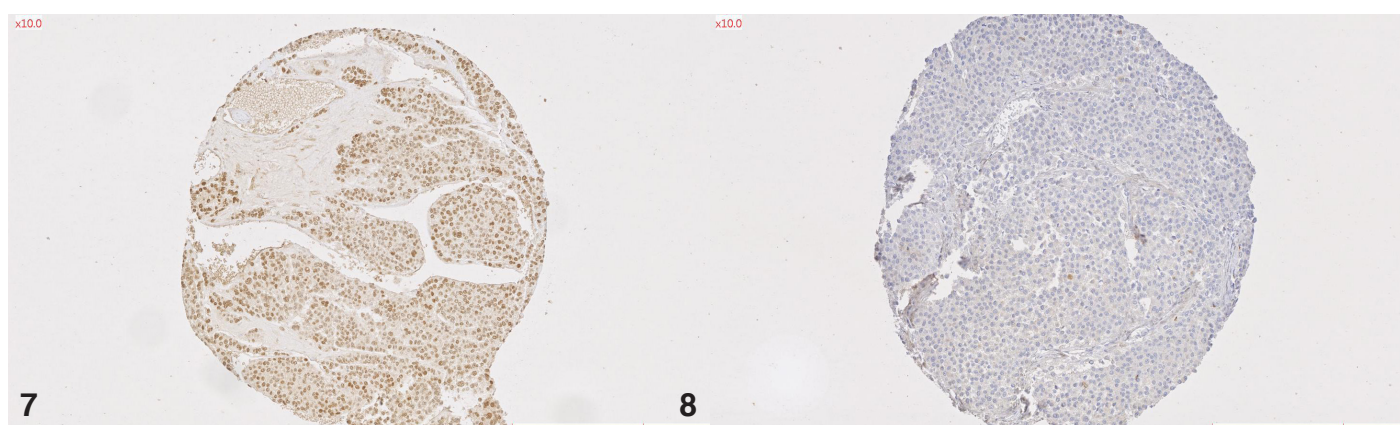

## GSTP1

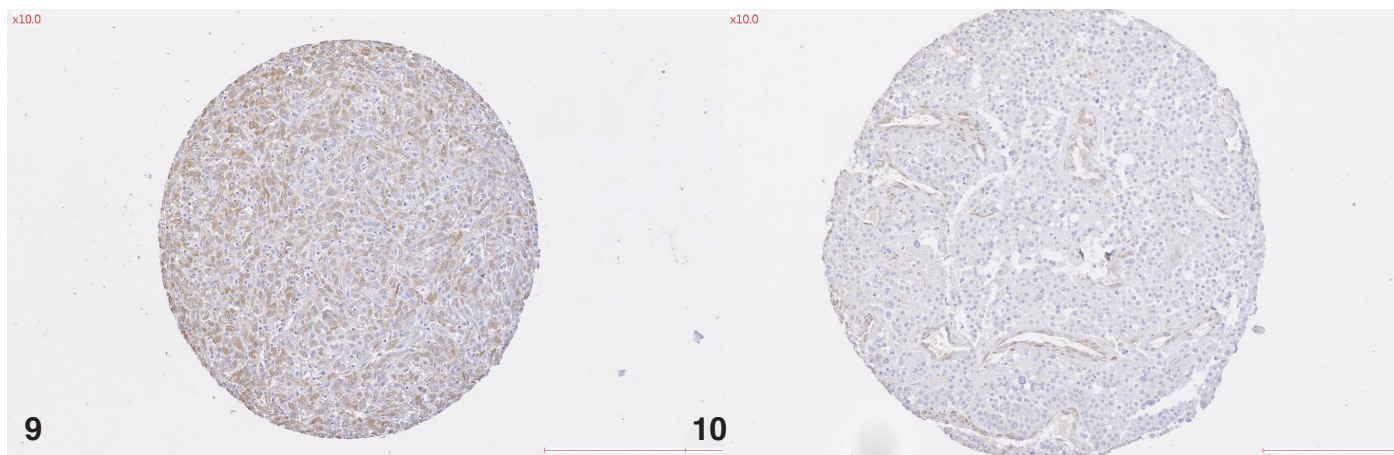

## Ki67

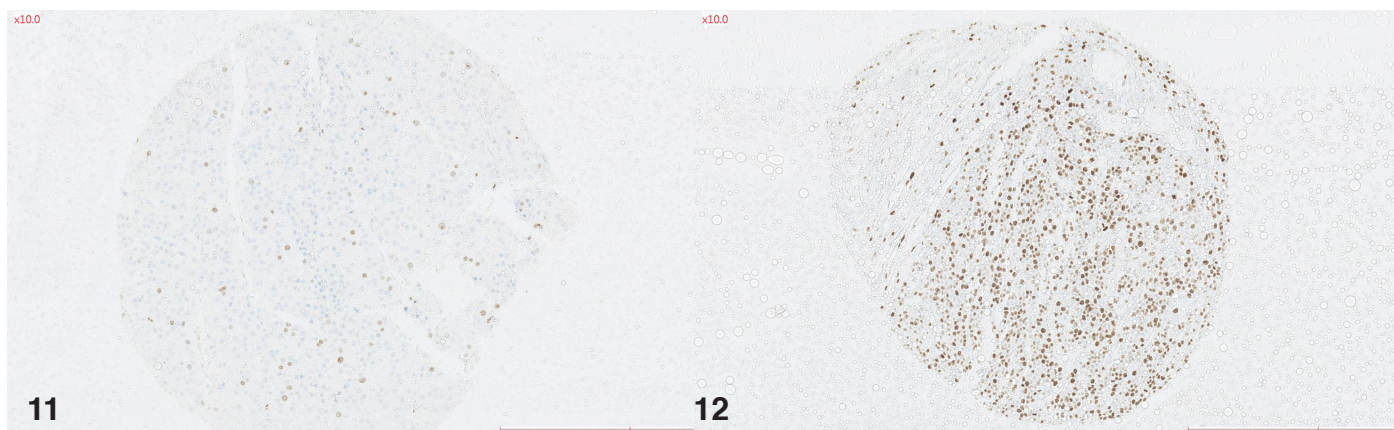

## LEF1

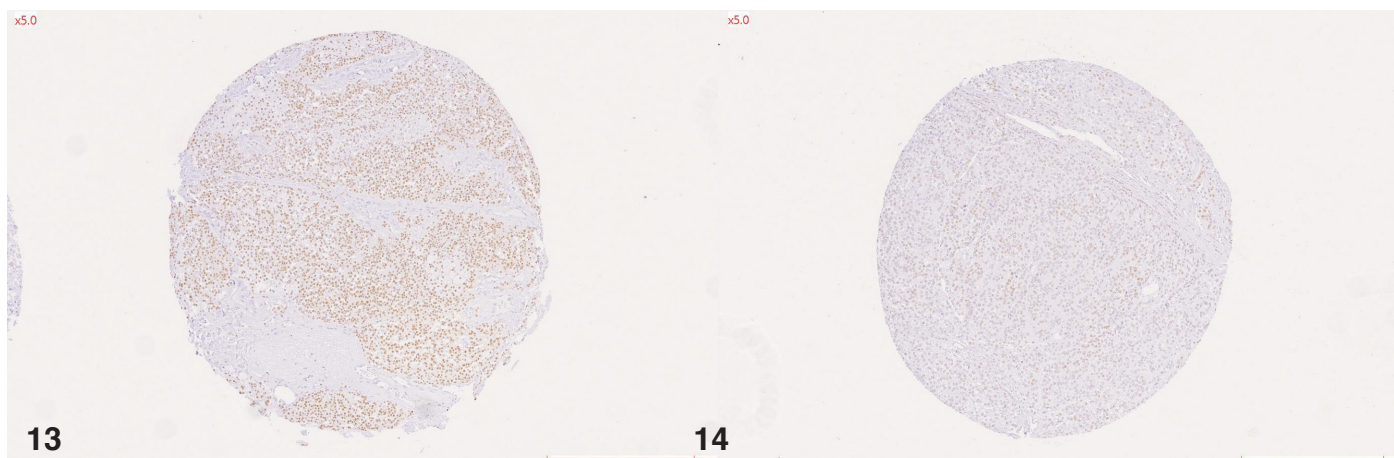

## MGMT

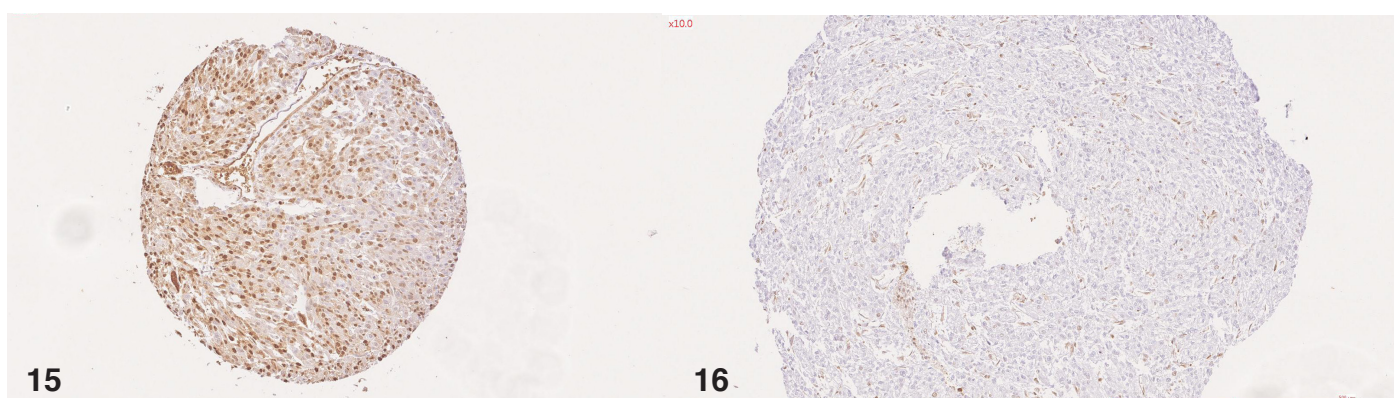

## P16

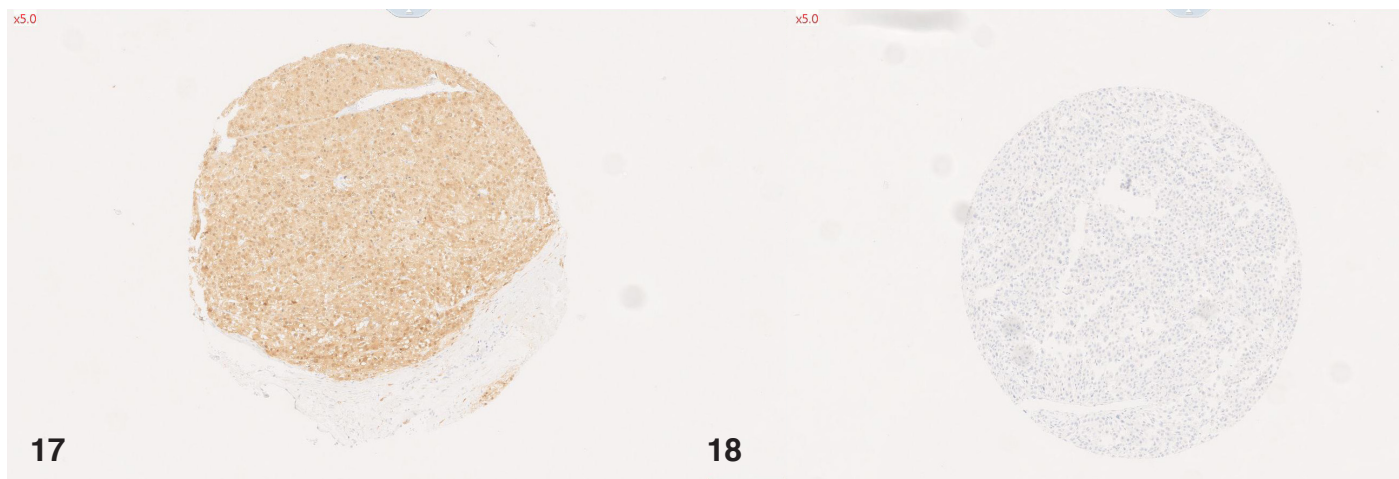

## P53

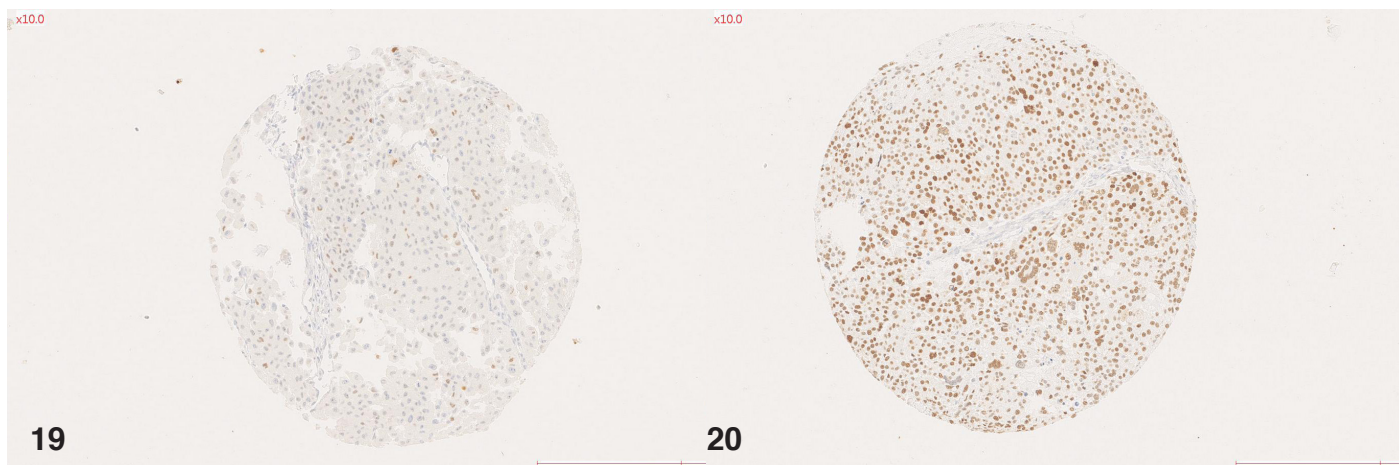

## PAX6

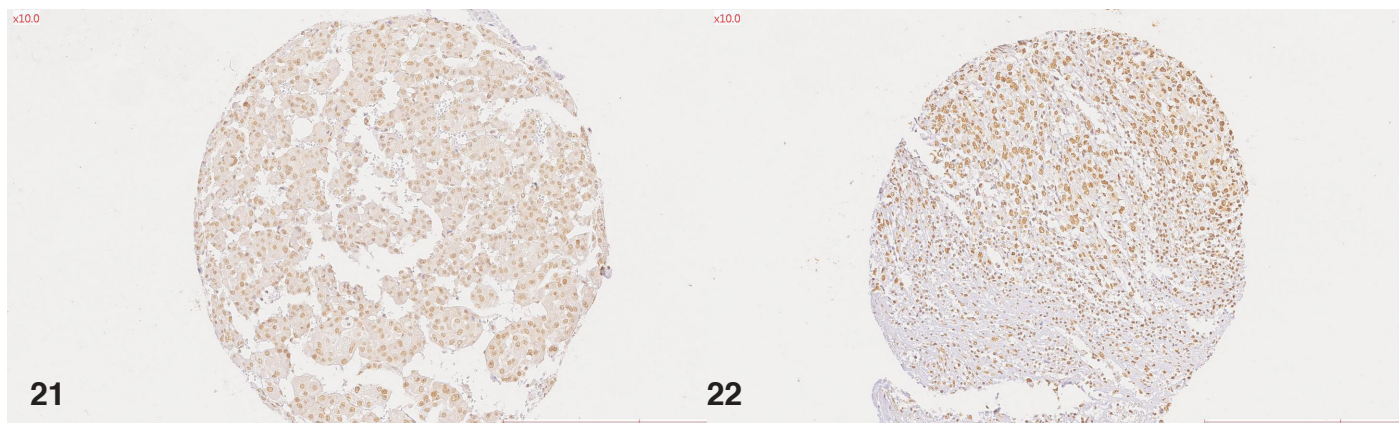

## RB

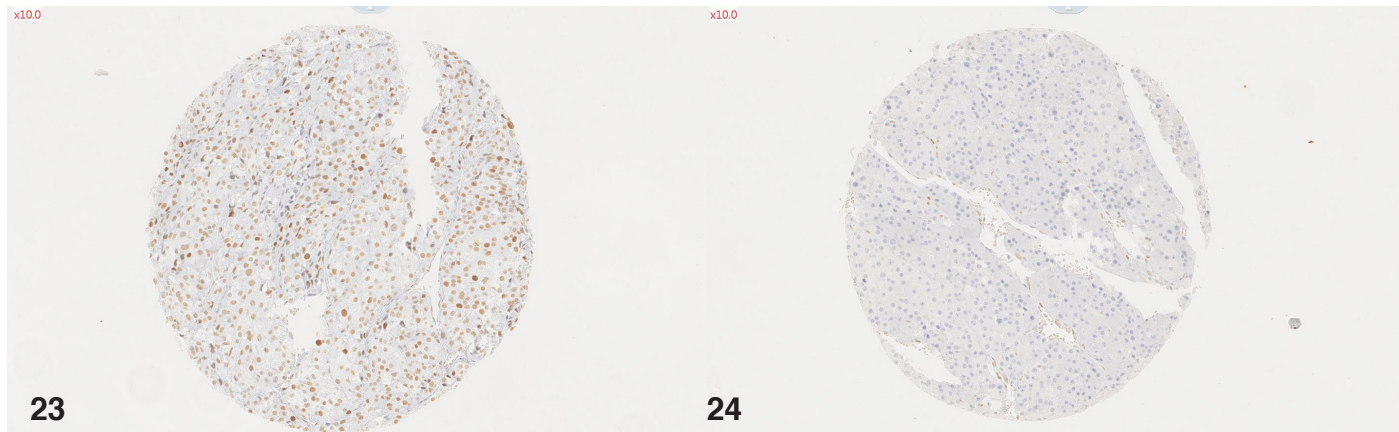

## RMM1

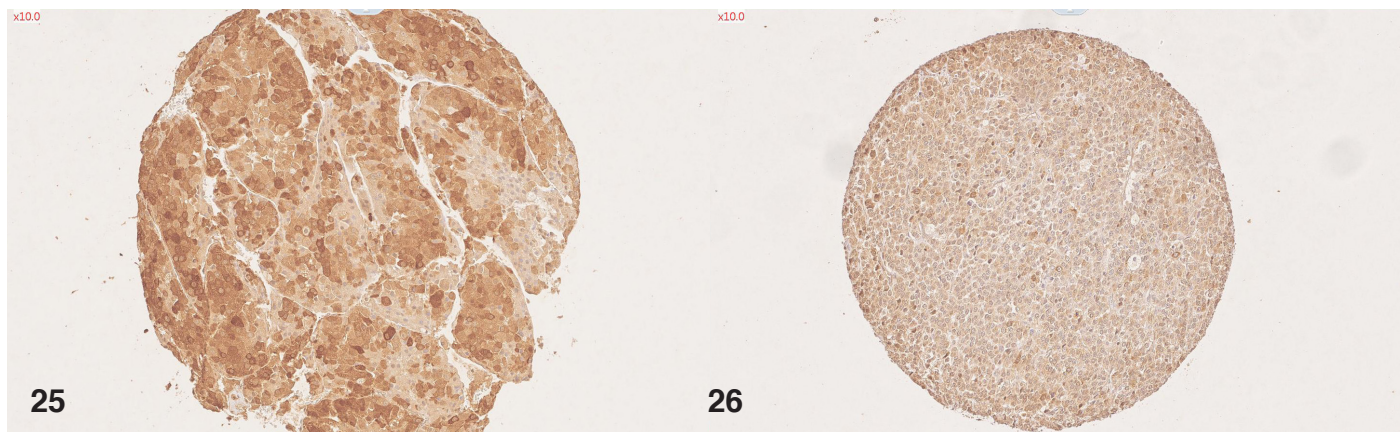

## SF1

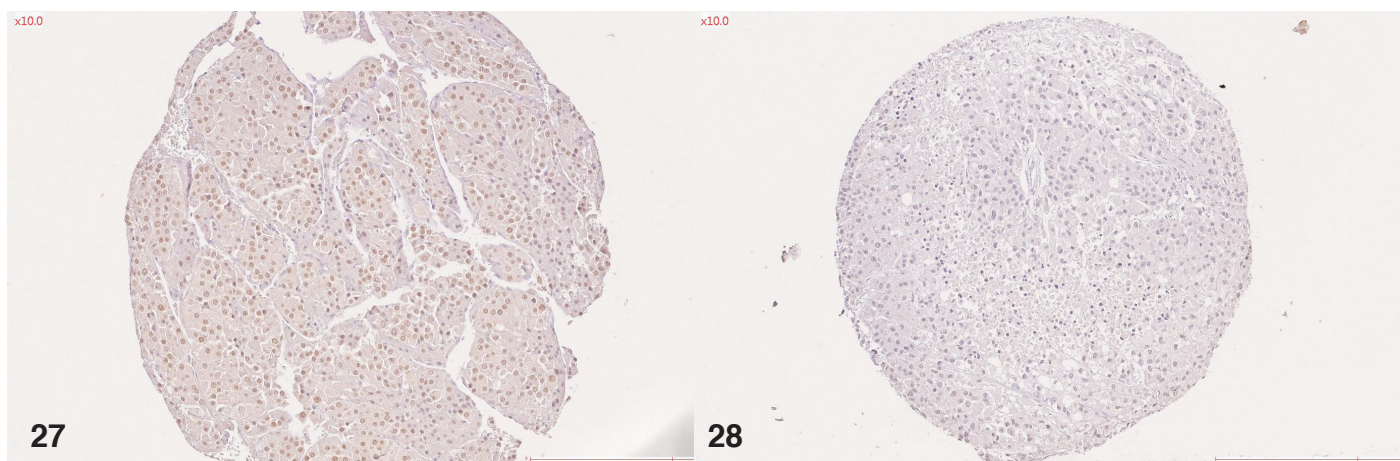

## SOAT1

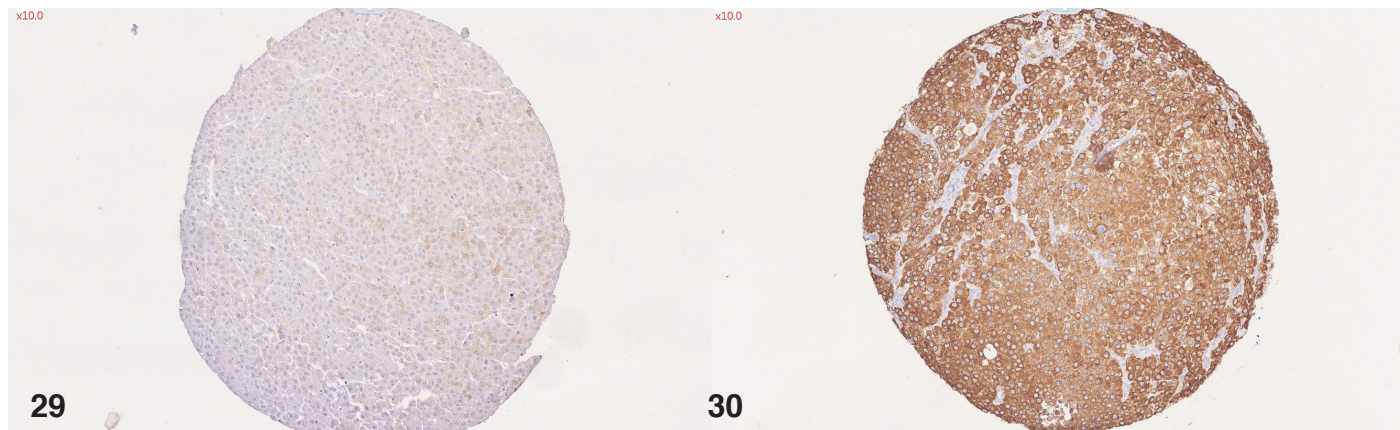

Supplement: Supplementary file 1 [file cancers-14-02225-s001.zip › supplementary material-final/Figure S1.pdf]
